# Hypertension, Diabetes and Overweight: Looming Legacies of the Biafran Famine

**DOI:** 10.1371/journal.pone.0013582

**Published:** 2010-10-22

**Authors:** Martin Hult, Per Tornhammar, Peter Ueda, Charles Chima, Anna-Karin Edstedt Bonamy, Benjamin Ozumba, Mikael Norman

**Affiliations:** 1 Department of Clinical Science, Intervention and Technology, Karolinska Institute, Stockholm, Sweden; 2 Department of Women's and Children's Health, Karolinska Institute, Stockholm, Sweden; 3 University of Nigeria Teaching Hospital, Enugu, Nigeria; Universidad Peruana Cayetano Heredia, Peru

## Abstract

**Background:**

Sub-Saharan Africa is facing rapidly increasing prevalences of cardiovascular disease, obesity, diabetes and hypertension. Previous and ongoing undernutrition among pregnant women may contribute to this development as suggested by epidemiological studies from high income countries linking undernutrition in fetal life with increased burden of non-communicable diseases in later life. We undertook to study the risks for hypertension, glucose intolerance and overweight forty years after fetal exposure to famine afflicted Biafra during the Nigerian civil war (1967–1970).

**Methods and Findings:**

Cohort study performed in June 27–July 31, 2009 in Enugu, Nigeria. Adults (n = 1,339) born before (1965–67), during (1968–January 1970), or after (1971–73) the years of famine were included. Blood pressure (BP), random plasma glucose (p-glucose) and anthropometrics, as well as prevalence of hypertension (BP>140/90 mmHg), impaired glucose tolerance (IGT; p-glucose 7.8–11.0 mmol/l), diabetes (DM; p-glucose ≥11.1 mmol/l), or overweight (BMI>25 kg/m^2^) were compared between the three groups. Fetal-infant exposure to famine was associated with elevated systolic (+7 mmHg; p<0.001) and diastolic (+5 mmHg; p<0.001) BP, increased p-glucose (+0.3 mmol/L; p<0.05) and waist circumference (+3cm, p<0.001), increased risk of systolic hypertension (adjusted OR 2.87; 95% CI 1.90–4.34), IGT (OR 1.65; 95% CI 1.02–2.69) and overweight (OR 1.41; 95% CI 1.03–1.93) as compared to people born after the famine. Limitations of this study include the lack of birth weight data and the inability to separate effects of fetal and infant famine.

**Conclusions:**

Fetal and infant undernutrition is associated with significantly increased risk of hypertension and impaired glucose tolerance in 40-year-old Nigerians. Prevention of undernutrition during pregnancy and in infancy should therefore be given high priority in health, education, and economic agendas.

## Introduction

Sub-Saharan African countries are in a process of a rapid epidemiological transition, away from infectious diseases, towards non-communicable diseases as leading causes of death [1]. Such transition in disease pattern is generally attributed to rural-to-urban shifts in adult lifestyle and typically involves changes in diet, cigarette smoking and lack of exercise [2]. There is a growing body of evidence however, suggesting that increased susceptibility to chronic diseases in adulthood has a developmental basis, originating in fetal life [3], [4]. The rapid development and shaping of the phenotype that occurs *in utero* is highly sensitive to environmental - in particular, nutritional – perturbations, leading to reduced functional capacity, altered metabolism and hormone production [3], [5]. This is considered an important underlying mechanism [3], [4] explaining why adults born small – a proxy for fetal starvation - are at increased risks for cardiovascular diseases and diabetes [6], [7].

The role of undernutrition during gestation for future health in children and adults has previously been evaluated in different countries [8]–[24]. However, besides data from European birth cohorts from the second world war [9]–[12], [19], [21]–[24], there is very limited information on the effects of fetal famine from other parts of the world, and with a follow-up time reaching beyond young adulthood. In particular, there is a lack of long-term follow-up data from sub-Saharan Africa, a continent with an unresolved situation with ongoing maternal-infant undernutrition, a recent and quick introduction of obesity-enhancing lifestyle factors and a rapidly increasing prevalence of cardiovascular disease, diabetes and hypertension [1], [25]–[27]. Therefore, the strength of a link between poor fetal nutrition and later adult diseases, would be of great interest to clarify in this part of the world also.

The study aim was to determine the risks for hypertension, diabetes and overweight forty years after fetal and infant exposure to the famine hitting Biafra during the Nigerian civil war (1967–1970) [28].

## Methods

This observational study is based on the findings from a cohort of 1,339 subjects, belonging to the ethnic group Igbo, who were born between 1965 and 1973, i.e., before, during and after the Biafran famine.

### Ethics statement

Ethical approval for the study was given by the Ethics Committee of the University of Nigeria Teaching Hospital, Enugu, and the Regional Ethical Vetting Board, Stockholm, Sweden. Informed consent (verbal and written in English, translated to Igbo if necessary) was obtained from all participants.

### The Nigerian civil war and the Biafran famine

The Nigerian civil war broke out on 6 July 1967, after the Igbo people in the south-eastern provinces had declared independence as the Republic of Biafra. The war was a culmination of ethnic, economic, and religious tensions among the various peoples of Nigeria. Disapproving of the secession, Nigerian forces rapidly pushed the Biafrans back into a small enclave. Inflow of food to this enclave was cut off. The result was extensive famine among the Igbos, regarded as one of the great nutritional disasters of modern times [28]. The war ended on 15 January 1970.

Of the 1 to 3 million Igbos that are estimated to have lost their lives, only a small fraction (10%) died of military violence. The majority succumbed to starvation. The nutritional emergency was most critical in the Biafran enclave, in which approximately 7 million people - mostly refugees - resided. In August 1968 the first international relief operations were launched but the amount of food provided was clearly not sufficient and the great majority of the Igbos did not get access to this relief food [29].

### Setting and participants

This follow-up study took place between June 27 and July 31, 2009. All shops at the six major market places of Enugu, the former Biafran capital, were systematically covered. People at the markets were actively contacted at their work place. The selection of participants was restricted to men and non-pregnant women who knew they were born in the southeast of Nigeria between 1965 and 1973. All subjects reported year of birth and 73% reported at least month and year. To confirm the birth year specified by the subject, we asked each person for a short review of his or her family history during the Nigerian civil war. The vast majority (more than 90%) of eligible subjects accepted participation in the study. It was not possible to do a systematic categorization of those who declined.

Data collection was conducted by MH, PT, PU, as well as doctors and medical students from the University of Nigeria Teaching Hospital (please see acknowledgements). Recruitment was performed by all team members, whereas measurements of blood pressure and p-glucose were obtained by a smaller group (n = 7) of specially trained investigators. Subjects were asked about level of education, current smoking, previously diagnosed hypertension or diabetes mellitus, and treatment for these conditions. Level of education was categorized as none, primary, secondary or higher education. There was no available information on birth weight or infant nutrition.

All measurements were performed according to predefined standard operating procedures. Participants were instructed to rest seated for at least five minutes before blood pressure (BP) and heart rate were measured. Two readings were taken three minutes apart in the left arm using a validated [30] automated oscillometric device (Omron M6 HEM-7001-E, Omron Corporation, Kyoto, Japan). A third reading was taken if the first and second BP differed more than five mmHg and mean systolic and diastolic values were calculated and defined as the subject's BP. Subjects having a systolic BP (SBP)≥140 mmHg, or a diastolic BP (DBP)≥90mmHg or who had a normal blood pressure but were pharmacologically treated for hypertension (n = 41 of 1,339), were categorized as hypertensive. Subjects having a SBP≥160 mmHg, and a DBP≥100 mmHg, were categorized as severely hypertensive. Random plasma glucose (p-glucose) was measured with a minimally invasive sampling technique (Abbott Freestyle *Lite*, Abbott Diabetes Care Ltd, Oxon, UK). A random p-glucose of 11.1 mmol/l or higher was classified as diabetes mellitus and impaired glucose tolerance (IGT) was defined as random p-glucose between 7.8 and 11.0 mmol/l [31]. A subgroup (n = 75) reported no food intake for at least 8 hours prior to investigation. In this group IGT was defined as p-glucose between 6.1 and 6.9 mmol/l and diabetes mellitus as a p-glucose ≥7.0 mmol/l [31]. Height and weight were obtained from all subjects using height sticks and digital scales, respectively, and body mass index (BMI) was calculated. Overweight was defined as a BMI over 25 kg/m^2^ and obesity as BMI over 30 kg/m^2^. Waist circumference was measured with a measuring tape placed around the abdomen at the level of the upper hip bone, and considered as an indicator of central obesity.

All participants were briefly counselled about their current health status. Hypertensive or glucose intolerant subjects were referred to the University of Nigeria Teaching Hospital for further investigation and treatment.

### Categorization according to exposure to famine

Subjects born between 1965 and 1967 were categorized as being exposed to famine in early childhood. Subjects born between 1968 and January 1970 (end of war) were categorized as being exposed to famine in fetal life and in infancy. Subjects born immediately after the surrender of Biafra were left uncategorized because of uncertainties in their exposure to famine and local variations in nutritional relief. Accordingly, subjects born between 1971 and 1973 and after the transitional period were categorized as being unexposed to famine.

Efforts to limit any potential bias included investigator-driven recruitment of participants (in contrast to subjects actively seeking participation in the study), direct assessments of outcomes with non-operator dependent methods and predefined standardized operational procedures applied by all data collectors.

### Statistical methods

We aimed to include 630 individuals in each group, based on an assumption of a standard deviation of 19 mmHg and a group difference in systolic blood pressure of 3 mmHg or more, given 80% power and a statistical significance level of 0.05. Because of exhausted recruitment at the six study markets, data collection was ended when 1,339 subjects had been included in the study. All subjects were included in logistic regression analyses according to year of birth whereas in group wise logistic regression analyses, the 173 subjects born during the transition from famine to nutritional relief, i.e., from February to December 1970, were excluded.

Data are presented as means (SD), proportions (%) and odds ratios (95% CI). Group differences were tested using ANOVA and chi-squared test. Logistic and linear regression analyses were used to calculate odds ratios (OR) and for evaluation of relations between exposure (fetal-infant famine), potential confounders (sex, smoking, educational level) and outcomes (blood pressure, p-glucose, and BMI). As several previous reports have shown that the associations between early life exposures and adult outcomes vary in relation to sex and adult body size [9], [10], [14], [22], [32]–[34], we also stratified our analyses on sex and BMI. A p-value <0.05 was considered as statistically significant. Analyses were performed using STATA/IC 11.0 (Stata Corp LP, TX, US). To evaluate gender differences analyses stratified according to sex were also performed.

## Results

Age differed among the three groups due to inclusion criteria. The proportion of men varied between 66 and 74% in the three groups and was highest in the group born after the famine. Smoking occurred predominantly in men and the proportion of smokers differed in the three groups, Table 1. Educational levels were found to be distributed equally between groups (p = 0.38) and overall, no education was found in 1%, primary in 34%, secondary in 52% and higher education in 13%.

**Table 1:** Subject characteristics.

	Born 1965–1967	Born 1968–	Born 1971–1973		*Feb–Dec 1970*
	Famine in	January 1970	Unexposed	P-value	*Transitional*
	early childhood	Fetal-infant famine			*period*
**Number of subjects (1,339)**	388	292	486		*173*
Sex, male	246 (66%)	189 (66%)	353 (74%)	0.01	*112 (65%)*
Age	43.0 (0.8)	40.5 (0.6)	37.0 (0.8)	N/A	*39*
Smoking, yes (%)	46 (12%)	27 (9%)	81 (17%)	0.007	*19 (11%)*
**Education, N (%)**					
None	6 (2%)	4 (1%)	5 (1%)	0,38	*4 (2%)*
Primary	138 (38%)	80 (30%)	152 (33%)		*46 (28%)*
Secondary	172 (48%)	152 (57%)	239 (52%)		*97 (58%)*
Higher	44 (12%)	33 (12%)	60 (13%)		*19 (11%)*
**Current data**					
Systolic blood pressure, mm Hg	125 (17)	129 (19)	122(16)	<0.0001	*124 (19)*
Diastolic blood pressure, mm Hg	81 (11)	84 (12)	79 (11)	<0.0001	*81 (12)*
Heart rate, bpm	75 (11)	76 (11)	75 (11)	0.27	*77 (10)*
Random p-glucose, mmol/l	6.1 (1.6)	6.4 (2.0)	6.1 (1.8)	0.04	*6.3 (2.6)*
Weight, kg	76.2 (12.9)	78.5 (13.4)	77.0 (13.4)	0.08	*76.3 (15.4)*
Height, cm	169 (8)	169 (8)	170 (8)	0.034	*169 (8)*
Waist circumference, cm	93 (11)	94 (13)	91 (11)	0.0011	*92 (12)*
BMI, kg/m^2^	26.7 (4.7)	27.5 (4.6)	26.5 (4.4)	0.016	*26.6 (5.1)*

Data are mean (SD) or number (%) of subjects. p-values for ANOVA or chi-squared test across groups. Data from transitional period (February 1970–December 1970) not included in the analyses. BMI = body mass index.

### Famine and risk for hypertension

SBP and DBP were higher after fetal-infant exposure to famine, as compared to the other two groups, i.e., those exposed in early childhood as well as those that were born after the famine, i.e., were unexposed, Table 1. In addition, the OR's for a SBP and DBP in the hypertensive range were significantly higher for the group exposed to fetal-infant famine, Table 2.

**Table 2:** Odds ratios for hypertension, impaired glucose tolerance, diabetes, overweight (BMI>25 kg/m2) and obesity (BMI>30kg/m2) in 40-year-old men and women exposed to famine in early childhood, fetal-infant life, or unexposed.

			ALL SUBJECTS				FEMALE				MALE			
			N = 1,166				N = 378				N = 788			
	N	n (%)	Crude		Adjusted for BMI		Crude		Adjusted for BMI		Crude		Adjusted for BMI	
			OR	C.I.	OR	C.I.	OR	C.I.	OR	C.I.	OR	C.I.	OR	C.I.
**SBP≥140**	1,166	177 (15)												
Childhood	388	61 (16)	1.78	(1.19–2.68)	1.77	(1.17–2.68)	1.45	(0.65–3.28)	1.46	(0.65–3.30)	2.04	(1.26–3.31)	2.11	(1.29–3.45)
Fetal-infant	292	70 (24)	3.02	(2.01–4.52)	2.87	(1.9–4.34)	3.52	(1.63–7.60)	3.47	(1.6–7.51)	2.85	(1.75–4.64)	2.72	(1.65–4.49)
Unexposed	486	46 (9.5)	1	Ref	1	ref	1	ref	1	ref	1	ref	1	ref
**DBP≥90**	1,166	204 (18)												
Childhood	388	64(16)	1.35	(0.93–1.97)	1.30	(0.88–1.91)	1.28	(0.65–2.51)	1.20	(0.61–2.38)	1.40	(0.87–2.23)	1.42	(0.88–2.29)
Fetal-infant	292	78 (27)	2.49	(1.72–3.62)	2.28	(1.56–3.34)	2.86	(1.48–5.52)	2.72	(1.40–5.27)	2.25	(1.41–3.58)	2.06	(1.27–3.33)
Unexposed	486	62 (13)	1	Ref	1	ref	1	ref	1	ref	1	ref	1	ref
**Severe HT** *	1,166	47 (4.0)												
Childhood	388	14 (3.6)	1.36	(0.63–2.93)	1.42	(0.65–3.13)	1.21	(0.32–4.61)	1.20	(0.31–4.59)	1.28	(0.49–3.38)	1.48	(0.54–4.03)
Fetal-infant	292	20 (6.8)	2.68	(1.31–5.46)	2.50	(1.19–5.26)	2.30	(0.65–8.10)	2.20	(0.62–7.79)	2.82	(1.18–6.73)	2.66	(1.05–6.73)
Unexposed	486	13 (2.7)	1	Ref	1	ref	1	ref	1	ref	1	ref	1	ref
**IGT**	1,114	108 (9.7)												
Childhood	374	34 (9.1)	1.15	(0.70–1.86)	1.13	(0.69–1.83)	0.82	(0.35–1.95)	0.79	(0.33–1.90)	1.33	(0.73–2.44)	1.32	(0.72–2.42)
Fetal-infant	278	37 (13)	1.76	(1.09–2.85)	1.65	(1.02–2.69)	1.23	(0.52–2.89)	1.12	(0.47–2.66)	2.02	(1.11–3.67)	1.93	(1.05–3.52)
Unexposed	461	37 (8.0)	1	Ref	1	ref	1	ref	1	ref	1	ref	1	ref
**Diabetes**	1,114	25 (2.2)												
Childhood	374	9 (2.4)	1.88	(0.66–5.33)	1.81	(0.64–5.15)	1.55	(0.36–6.64)	1.49	(0.35–6.44)	1.93	(0.43–8.68)	1.92	(0.43–8.69)
Fetal-infant	279	11(3.9)	3.11	(1.14–8.51)	2.56	(0.92–7.17)	2.06	(0.48–8.86)	1.85	(0.43–8.03)	3.84	(0.95–15.5)	3.15	(0.74–13.4)
Unexposed	461	6 (1.3)	1	Ref	1	ref	1	ref	1	ref	1	ref	1	ref
**Overweight**	1,150	732 (64)												
Childhood	384	238 (62)	1.02	(0.77–1.34)			1.32	(0.72–2.42)			0.82	(0.59–1.14)		
Fetal-infant	287	199 (69)	1.41	(1.03–1.93)			2.13	(1.04–4.35)			1.19	(0.83–1.71)		
Unexposed	479	295 (62)	1	Ref			1	ref			1	ref		
**Obesity**	1,150	253 (22)												
Childhood	384	88 (23)	1.20	(0.87–1.67)			0.95	(0.57–1.58)			1.11	(0.69–1.80)		
Fetal-infant	287	70 (24)	1.30	(0.92–1.85)			1.23	(0.72–2.12)			1.03	(0.60–1.75)		
Unexposed	479	95 (20)	1	Ref			1	ref			1	ref		

SBP = systolic blood pressure, DBP = diastolic blood pressure, HT = hypertension, IGT = impaired glucose tolerance.

*SBP≥160 and DBP≥100 mmHg.

In linear regression analyses, SBP and DBP were associated with BMI (β = 0.49, r = 0.13, p<0.001 and β = 0.54, r = 0.21, p<0.001, respectively) and male sex (β = 3.3, r = 0.08, p = 0.003 and β = −1.46, r = 0.06, p = 0.05, respectively), but not with smoking. After adjusting for BMI odds ratios for SBP and DBP in the hypertensive range were of the same magnitude as before adjustment and still significantly higher for the group exposed to fetal-infant famine. In addition, analyses stratified on sex showed that exposure to famine in fetal life and infancy was a risk factor for SBP and DBP in the hypertensive range in both women and men. The OR (95% CI) for severe hypertension in the group exposed to fetal-infant famine was 2.82 (1.18–6.73) in men and 2.30 (0.65–8.10) in women, Table 2.

**Figure 1:**
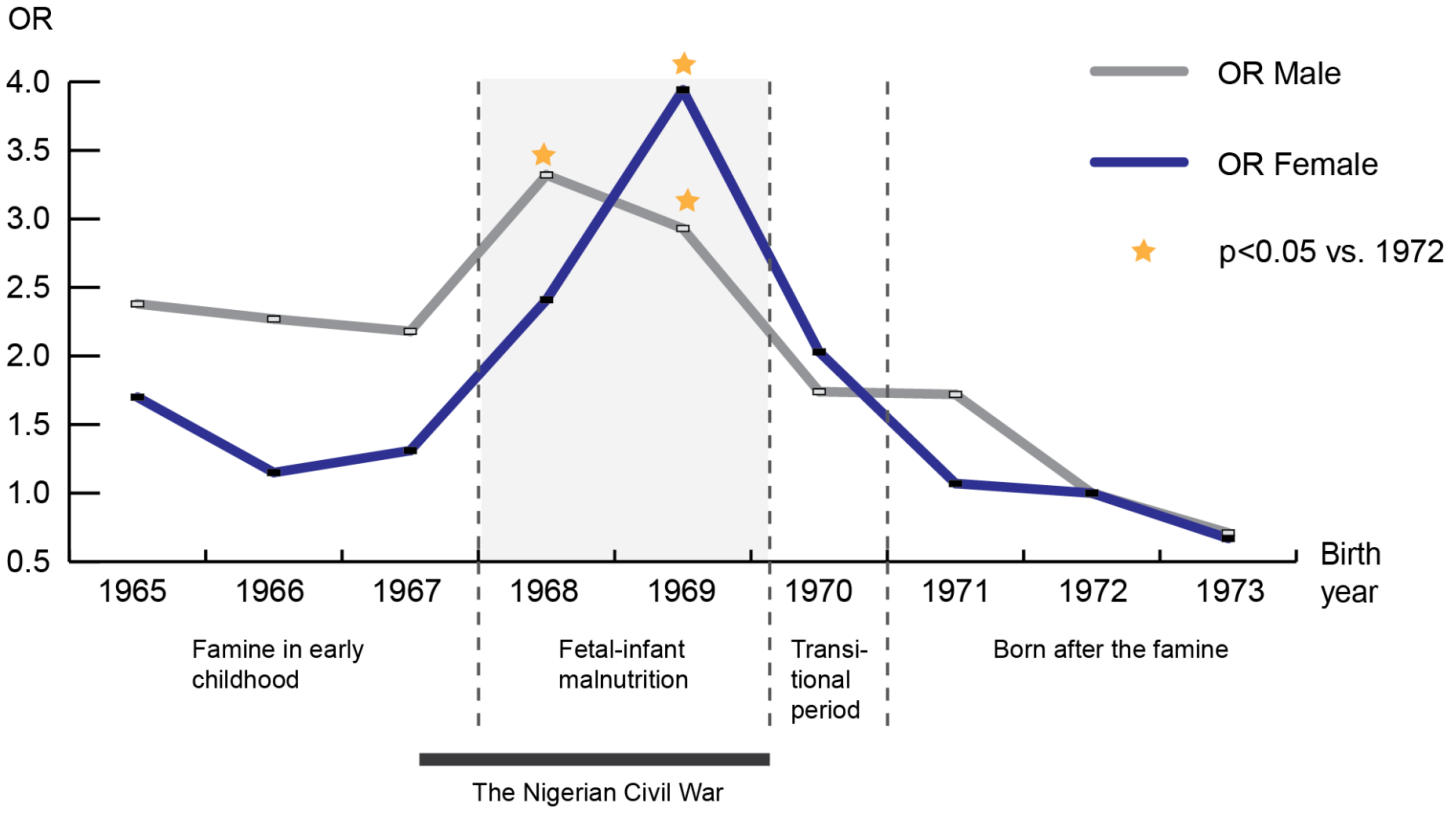
Odds ratios for high systolic (≥140 mmHg) blood pressure in men and women at follow-up in 2009 according to year of birth and with 1972 as reference.

Odds ratios for high SBP (≥140 mmHg) according to each year of birth and with 1972 as reference year (OR = 1), are presented in Figure 1. The figure shows that famine in fetal-infant life (birth-year 1968 and 1969) resulted in a three to four fold increase in risk for an adult SBP in the hypertensive range, both in women and men.

Odds ratios for high SBP (≥140 mmHg) in relation to exposure group (childhood famine, fetal-infant famine and unexposed) and stratified on current overweight (BMI≥25), are presented in Table 3. Formal statistical testing for an interaction between early exposure to famine by sex and adult overweight was not significant, suggesting that the effects of early famine and current overweight on SBP were additive to each other.

**Table 3:** Odds ratios (OR) for systolic blood pressure (SBP)≥140 mm Hg and impaired glucose tolerance stratified on exposure to early famine and current BMI.

		GROUP					
		Childhood Famine		Fetal-Infant Famine		Unexposed	
	BMI	OR	95% C.I.	OR	95% C.I.	OR	95% C.I.
**SBP≥140 mm Hg**	≤25	2.14	(0.88–5.45)	2.49	(0.91–6.81)	1	Ref
	>25	3.95	(1.88–9.04)	6.98	(3.36–15.84)	2.34	(1.10–5.44)
**Impaired glucose tolerance**	≤25	2.58	(0.94–7.77)	3.43	(1.16–10.48	1	ref
	>25	2.23	(0.88–6.39)	3.57	(1.45–10.02)	2.78	(1.16–7.66)

### Famine and risk for impaired glucose tolerance/diabetes

Random p-glucose was higher in adults exposed to famine in fetal-infant life, Table 1. The crude odds ratio for both IGT and diabetes was significantly higher for the group exposed to fetal-infant famine in comparison to the subjects born after the famine. Exposure to famine in early childhood did not increase the risk for IGT and diabetes in later life, Table 2.

In linear regression analyses, random p-glucose was associated with BMI (β = 0.04, r = 0.12, p<0.001) and male sex (β = −0.41, r = 0.10, p = 0.001). In analyses adjusted for BMI, fetal-infant exposure to famine remained as a risk factor for IGT and diabetes in Nigerian subjects in their forties. Stratified analyses on sex showed that the OR (95% CI) for diabetes after exposure to fetal-infant famine was 3.84 (0.95–15.5) in men and 2.06 (0.48–8.86) in women, Table 2.

### Famine and risk for overweight

The group born after the famine was significantly taller than the other two groups, whereas there were no group differences in weight, Table 1. Waist circumference and BMI were higher in adults exposed to famine in fetal-infant life, Table 1. The crude odds ratio for overweight (BMI>25) was significantly higher for the group exposed to fetal-infant famine in comparison to the subjects born after the famine. Stratified analyses on sex showed that this effect was confined to women Table 2.

## Discussion

This study showed higher BP, higher p-glucose and higher weight in middle-aged Nigerian people exposed to severe undernutrition *in utero* and in infancy. Comparing unexposed offspring with that of starving pregnant women, fetal-infant undernutrition was associated with significant increases in the prevalence of hypertension (from 9.5 to 24%, defined as SBP≥140 mmHg) and impaired glucose tolerance or diabetes (from 8.0 to 13%). Famine in early childhood was also associated with an increased prevalence of adult blood pressure in the hypertensive range (from 9.5 to 16%). Given the additive effects of early famine and adult overweight (Table 3), early undernutrition followed by later overnutrition seem to provide two fundaments for the adverse metabolic and cardiovascular outcomes seen in today's Nigeria, and as also previously described in an Indian cohort (14).

Our results are in line with previous epidemiological and experimental studies suggesting that fetal undernutrition contributes significantly to cardiovascular disease risk in adult life [3], [5], [20], [35]. In contrast to observations in European birth cohorts [9]–[12], [19], [20]–[24], the effects of fetal undernutrition seem to be more pronounced and emerge at an earlier age in this sub-Saharan cohort. These differences could reflect variations in exposure and population differences. Such an explanation may not necessarily be confined to susceptibility for and degree of fetal-infant undernutrition alone. Accelerated growth in later childhood [22], [32] and a high BMI in adult life have previously been found to have a stronger adverse effect on hypertension [33], [34] and diabetes [9], [10] in people who were small at birth. The mismatch between the environment that people in urban Nigeria now live in, characterized by a high-calorie-high-carbohydrate diet, and the one within which they evolved during the Biafran famine, may therefore be largely responsible for the present increase in disease risks [36]. Our finding of a higher current weight and waist circumference, and a higher BMI in adults exposed to fetal-infant famine (Table 1) imply that accelerated growth later in childhood may be in the pathway between early under-nutrition and later metabolic syndrome. Such an explanation may also be valid for the finding that glucose tolerance and blood pressure in 35-year-old Gambians (predominantly women) who remained fit and lean, showed no association with moderate to severe malnutrition in early life [37]. However, as we found no attenuation of the excess odds for adult hypertension and impaired glucose tolerance after adjusting for current BMI (Table 2), the association between fetal-infant famine and adult metabolic syndrome cannot be attributed to accelerated childhood growth alone [38].

Gender differences in developmental programming have previously been described [32]. In our study, both men and women exposed to fetal-infant undernutrition exhibited higher odds ratios for elevated blood pressure at follow-up. An excess risk for IGT was also found in men whereas power limitations unable conclusions regarding the risk for IGT in women exposed to early famine. Conversely, a significant excess risk for overweight was only seen in women exposed to fetal-infant undernutrition. Although there are reports of increased susceptibility for fetal programming of BP in males [23], [32], sex differences in the association between birth weight and BP have been questioned [39]. Experimental data suggest possible sex specific mechanisms in fetal programming of insulin secretion and insulin resistance [40], mechanisms that may be relevant in explaining our findings of gender differences in glucose tolerance.

The strengths of this study include the design with prospectively set inclusion criteria and active enrolment of a large cohort of customers and traders at markets, i.e., places where many people in the urban areas gather and work, as other sectors of employment and sites for purchasing of everyday goods are not very developed in this part of the world. Thus we believe that the study cohort is representative for urban settings in sub-Saharan Africa and at highest risk for the present epidemic in non-communicable diseases. Categorization was based on date of birth – i.e., exposure to famine in early life. In addition, since subjects born in the transitional period were excluded, there was no late gestational overlap with famine in the unexposed group. The follow-up time was sufficiently long to establish relations to outcomes that are directly related to adult cardiovascular disease. Finally, we addressed the possibility that smoking confounded our results [41].

Although heritability for birth weight has been estimated to range from 25% to 40% [42], and although some experimental data suggest that birth weight may fail to reflect intrauterine factors associated with later disease risk [43], birth weight is the most commonly used proxy for fetal undernutrition. Therefore, a limitation of our study, shared with other famine studies [19], [23], is the lack of anthropometric data at birth and in infancy. There are no records from which these data can be retrieved. In addition, we have no data on mother and infant nutrition. Given that inflow of food to Biafra was cut off, it can be assumed that access to infant formula was extremely limited and that most infants were exclusively breastfed.

It is likely that survival of the healthiest pregnant women occurred during the Biafran famine. The most severe cases of fetal and infant undernutrition are also likely to have died prior to follow-up, either in early childhood or from the increasing cardiovascular morbidity reported from adults residing in the area [25]. Some misclassification may have occurred as the nutritional situation of Biafra gradually deteriorated, making it difficult to pinpoint the start of famine. If anything, these limitations may have introduced a conservative bias into our study, underestimating the true long-term effects of undernutrition in fetal life and in infancy.

Before the war, Biafra differed from most developing nations because it had a good supply of food and water, and sound public health policies with many physicians, nurses, hospitals, and clinics [44]. Already months after the war, there are reports of vast improvement in the nutritional situation [28]. Our results are therefore most likely reflecting differences in adult health outcomes after severe early famine as compared to a significantly better nutritional situation in early life.

The external validity of data from a convenience sample from market places can also be discussed. The method of recruiting participants will have missed subsistence farmers and others not attending the markets, whose nutritional status and other factors are likely to differ from market people. We note that the prevalences of hypertension, diabetes and obesity reported herein are similar to those reported from comparable urban and rural Nigerian cohorts [45], [46].

The study design, i.e., comparing long-term outcomes in people born before, during or after famine, has previously been used [9]–[12]. By inclusion, the subjects in the unexposed group were youngest. As the prevalence of hypertension and glucose intolerance increases with age, some associations with birth year might be expected. However, given the size of the effect and that the OR's for all outcomes after fetal-infant famine were significantly lower not only in younger, but also in older people, trends in disease-risks over time and cohort effects cannot be the only explanation for our findings.

Previous studies indicate that a nutritional insult - during gestation or the first few months of postnatal life - may be important for later outcome and disease risk [9], [11]. Although the resolution and exposure data of this study do not allow for a detailed analysis of the timing of the insult, the striking dose-response effect found between birth during years of famine and over-risk for hypertension in adult life (Figure 1) suggests a causal relationship. The Biafran famine was characterized by a severe scarcity of proteins, manifested in the vast number of infants and children suffering from kwashiorkor [28]. Experimental models suggest that protein deficit *in utero* may programme abnormal glucose homeostasis and vascular endothelial dysfunction, whereas results are less consistent with regard to programming of high blood pressure [35], [43]. Besides the nutritional insult, pregnant women in former Biafra were living under conditions of war. Such stress for mothers and infants could also contribute to higher blood pressure in later life [47].

The implications of our findings are important. On a population level, a 3.3 mmHg increase in mean SBP and 2 mmHg in DBP can be translated into an estimated increase in cardiovascular deaths by 25% and stroke by 32% [48]. Given the combination of large blood pressure effects and increased rates of glucose intolerance resting on a basis of prevalent obesity before middle age (Table 3), which is characteristic for Nigeria today, it is not surprising that disability and deaths from stroke and coronary heart disease are rapidly increasing. To adequately address this trend in non-communicable diseases on a community level requires a developed health care infrastructure providing life-long treatment and follow-up. The increasing burden of chronic diseases therefore poses a massive challenge to the already crippled health care systems of sub-Saharan Africa.

In summary, our study demonstrates a fetal-infant contribution to adult hypertension and glucose intolerance in an African cohort. It can be assumed that fetal and infant undernutrition is a significant contributor to the increasing prevalence of hypertension and glucose intolerance also in other parts of sub-Saharan Africa. Therefore, prevention of fetal and infant undernutrition should be given high priority in national health, education, and economic agendas to limit the increase of non-communicable diseases in many African countries. Given that the highest risk for hypertension was found in those undernourished in early life and then growing overweight, it is appropriate to consider that preventing excess growth in later childhood may be as important for reducing adult ill-health as supporting fetal-infant growth.
